# A fully feathered enantiornithine foot and wing fragment preserved in mid-Cretaceous Burmese amber

**DOI:** 10.1038/s41598-018-37427-4

**Published:** 2019-01-30

**Authors:** Lida Xing, Ryan C. McKellar, Jingmai K. O’Connor, Ming Bai, Kuowei Tseng, Luis M. Chiappe

**Affiliations:** 1 State Key Laboratory of Biogeology and Environmental Geology, China University of Geosciences, Beijing, 100083 China; 2 School of the Earth Sciences and Resources, China University of Geosciences, Beijing, 100083 China; 3 Royal Saskatchewan Museum, Regina, Saskatchewan S4P 4W7 Canada; 4 Biology Department, University of Regina, Regina, Saskatchewan S4S 0A2 Canada; 5 Department of Ecology & Evolutionary Biology, 1501 Crestline Drive – Suite 140, University of Kansas, Lawrence, Kansas 66045 USA; 6 Key Laboratory of Vertebrate Evolution and Human Origins of the Chinese Academy of Sciences, Institute of Vertebrate Paleontology and Paleoanthropology, Beijing, 100044 China; 7 Key Laboratory of Zoological Systematics and Evolution, Institute of Zoology, Chinese Academy of Sciences, Beijing, 100101 China; 8 Department of Exercise and Health Science, University of Taipei, Taipei, 11153 China; 9 Dinosaur Institute, Natural History Museum of Los Angeles County, Los Angeles, CA 90007 USA

## Abstract

Over the last three years, Burmese amber (~99 Ma, from Myanmar) has provided a series of immature enantiornithine skeletal remains preserved in varying developmental stages and degrees of completeness. These specimens have improved our knowledge based on compression fossils in Cretaceous sedimentary rocks, adding details of three-dimensional structure and soft tissues that are rarely preserved elsewhere. Here we describe a remarkably well-preserved foot, accompanied by part of the wing plumage. These body parts were likely dismembered, entering the resin due to predatory or scavenging behaviour by a larger animal. The new specimen preserves contour feathers on the pedal phalanges together with enigmatic scutellae scale filament (SSF) feathers on the foot, providing direct analogies to the plumage patterns observed in modern birds, and those cultivated through developmental manipulation studies. Ultimately, this connection may allow researchers to observe how filamentous dinosaur ‘protofeathers’ developed—testing theories using evolutionary holdovers in modern birds.

## Introduction

Burmese amber has been studied extensively for its arthropod and botanical inclusions^1,2^, but has only recently been recognized as a valuable source of diverse vertebrate material living during the beginning of the Cenomanian (98.8 ± 0.6 Ma)^3–7^. The amber deposit has been particularly informative for the integument of Enantiornithes^5,8,9^, the dominant clade of Cretaceous birds, supplementing our understanding of integumentary features otherwise only preserved in two-dimensional fossils from lacustrine sediments (e.g.^10–17^). Burmese enantiornithine remains preserved in amber contribute an unprecedented level of detail to our understanding of their plumage and soft tissues. Research on Burmese amber has united feathers in amber with skeletal material for the first time. To date, amber inclusions of enantiornithines with both skeletal material and feathers have been recovered for a pair of juvenile wing fragments^5^; a partial hatchling^8^; and the compacted skeleton of a juvenile^9^. Burmese amber specimens have exhibited a range of preservational qualities, extending from nearly pristine bone that retains its osteon microstructures (e.g., DIP-V-15100^5^, DIP-V-15102^9^), to material that has been dissolved and replaced by clay minerals (e.g., DIP-V-15103^6^). Inferred taphonomic pathways have varied, including encapsulation in resin while still alive (e.g., DIP-V-15100^5^), or partial encapsulation followed by significant weathering or scavenging (e.g., HPG-15-1^8^ and DIP-V-15102^9^).

**Figure 1:**
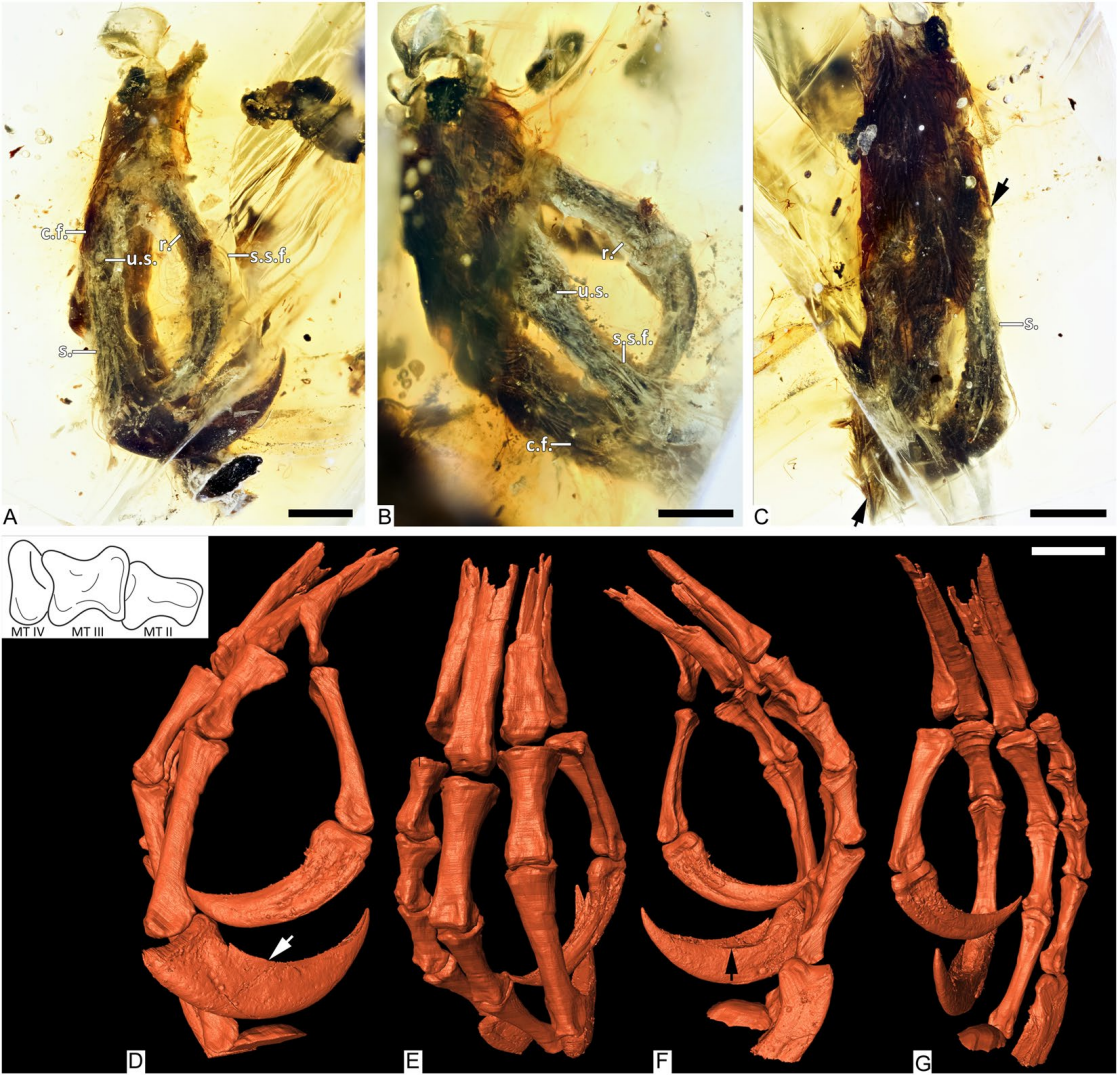
Overview and SR X-ray µCT renderings of dismembered foot, DIP-V-15105a. Foot soft tissue and plumage in (**A**) medial, (**B**) mediodorsal, and (**C**) dorsal views, with edge of contour feather coverage between arrows. Rendering of underlying bones in (**D**) medial, (**E**) mediodorsal, (**F**) lateral, and (**G**) plantar views. Arrows in D and F mark edge of keratin sheath, and one of its plantar ridges, respectively. Abbreviations: c.f., contour feather; r., reticulae; s., scute; s.s.f., scutellate scale filament; u.s., unfeathered scutellae. Scale bars = 1.0 mm.

**Figure 2:**
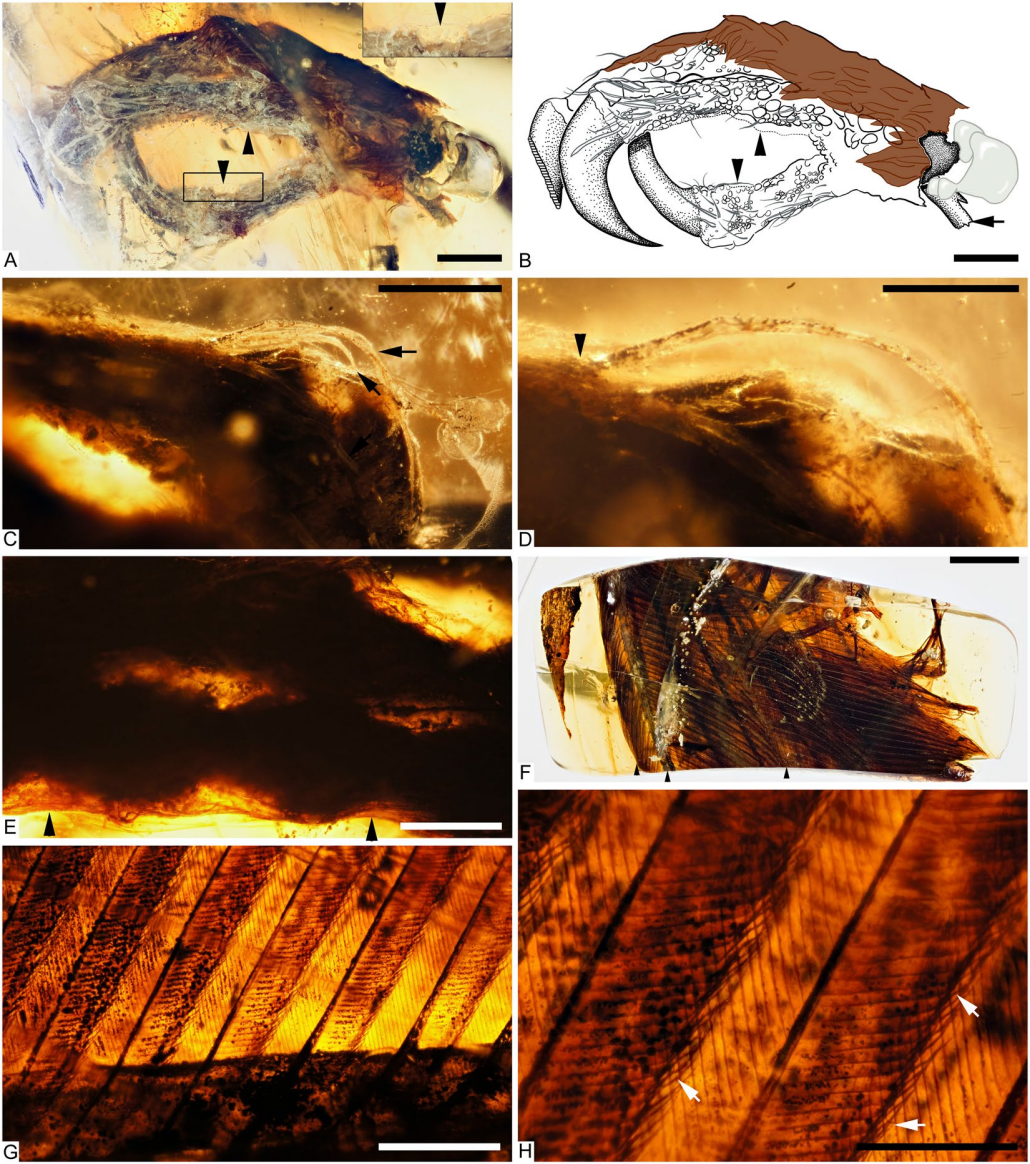
Plumage in DIP-V-15102 foot and wing. (**A–E**) DIP-V-15105a soft tissue and plumage; (**F–H**) DIP-V-15105b wing fragment plumage. **(A)** Medial view of foot, with oblique lighting to highlight integumentary structures. (**B**) Illustration of plumage types, and outlines of visible scutes, scutellae, and reticulae, corresponding to (**A**), indicating contour feathers inclined over outer toes (brown), broken ends of metatarsal (arrow), skin withdrawn from amber (arrowheads), gas bubble escaping from medullary cavity (grey), and carbonized soft tissue or insect frass (dark stipple). (**C**) SSFs (arrows) near ungual of digit II. (**D**) Higher magnification view of SSFs in (**C**), showing insertion into scute (arrowhead), and mottled appearance created by sporadic pyrite deposition. (**E**) Unfeathered apical portions of digits III and IV in medial view, with skin layer (between arrowheads) closely adhering to bones and bearing scutellae (see Fig. S2C). (**F**) Overview of overlapping primary sections from right wing, with arrowheads (from left to right) marking leading edge of P10, rachis of P10, and trailing edge of P10. (**G**) Barbs and rachis near base of primary P10. **(H)** Barbs and barbules of P10 in (**G**), with subtle hooklets (arrows). Scale bars = 1.0 mm (**A–C**,**E**); 0.25 mm (**D**,**H**); 3 mm (**F**); 0.5 mm (**G**).

**Figure 3:**
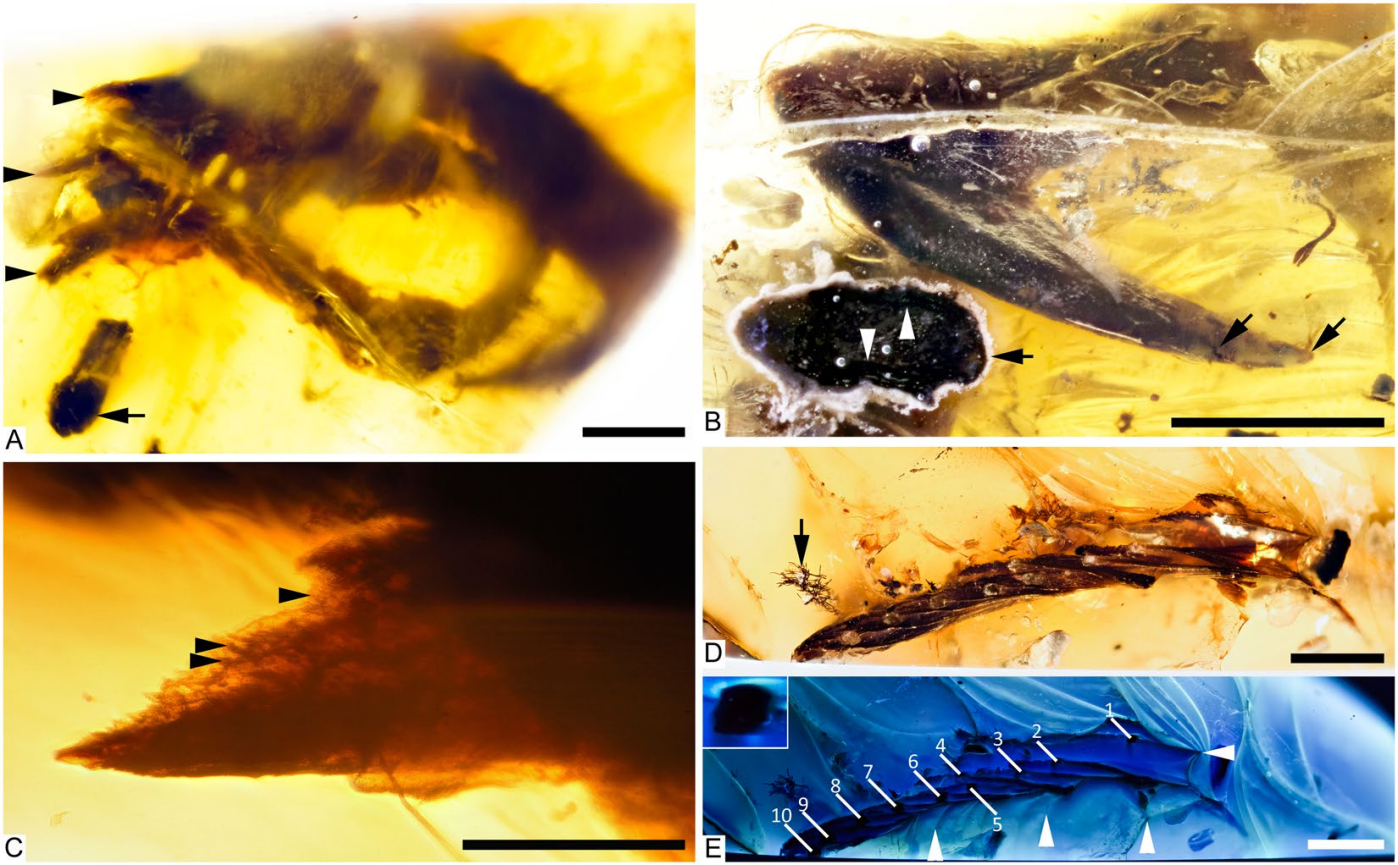
Taphonomy of DIP-V-15102 foot and wing. (**A–C**) DIP-V-15105a dismembered foot; (**D**,**E**) DIP-V-15105b wing fragment plumage. (**A**) Posterolateral view of foot, showing broken and displaced metatarsal fragments (arrowheads), and insect frass pellet (arrow). (**B**) unguals cross-cut by polishing of amber piece, with veil of milky amber (arrow), indistinct boundary (arrowhead) between black, carbonized ungual sheath and underlying bone with dark staining, and region of translucent ungual without underlying bone (between inclined arrows). (**C**) Detail of break at uppermost arrow in (**A**), displaying greenstick fracture pattern and traces of haversian canals within cortical bone (arrowheads). (**D**) Bases of primaries cross-cut by polishing of amber piece, leading edge of wing adjacent to clump of plant trichomes (arrow). (**E**) UV image of (**D**), displaying flow lines disrupted by primary feathers (arrowheads), rachises of P1–P10 (numbered lines), and D-shaped basal rachis cross section of P1 (insert). Scale bars = 1.0 mm (**A**,**B**); 0.25 mm (**C**); 2 mm (**D**,**E**).

Here we describe a partial foot with unusual feathering (DIP-V-15105a) that was found associated with the distal feathers from a partial wing tip (DIP-V-15015b). Preservation of bone structure is better in DIP-V-15105a than in most other enantiornithine inclusions reported to date. Unlike some previous discoveries, this inclusion provides bone preservation with the contrast necessary for detailed SR X-ray µCT osteological observations of most pedal elements; the individual bones have not been distorted by compaction^6,8,9^; and traces of a network of haversian canals are visible within the exposed bone ends (Figs 1, 3C). Feathers from both the foot and wing fragment (Fig. 2) provide some insight into their developmental stage, with the extensive contour plumage of the foot providing some clues about the overall appearance of the individual, including the first documentation of feathered pedal digits in a stem bird. The foot inclusion demonstrates a new plumage pattern for enantiornithines—one that has similarities to both modern birds and theropods basal to the Enantiornithes^18,19^.

## Results

### Osteological characters

The amber subsample preserves the distal third of a right tarsometatarsus, and the nearly complete pedal digits I–IV, missing only the distal portions of the ungual phalanges of digits III and IV (Fig. 1D–G). The metatarsals II-IV are hollow and completely unfused to one another. Metatarsals II and III are subequal in mediolateral width, whereas metatarsal IV is thinner and its distal trochlea is reduced to a single condyle, both features characteristic of many enantiornithines. Metatarsal III extends farther distally than metatarsals II and IV, which are nearly equal in distal extent with metatarsal IV ending slightly distal to metatarsal II. Metatarsals II and IV end approximately level with the proximal margin of the metatarsal III trochlea. Metatarsal I is J-shaped when viewed medially, a condition known for some enantiornithines^20^. This metatarsal is slightly disarticulated so that its deeply concave lateral surface is partially visible; this surface forms a tight articulation with the medial surface of metatarsal II. The proximal half of the metatarsal is thin and tapered to a point. The distal half is expanded and oriented plantodistally so that the shaft and straight plantar surface of the trochlear process define an obtuse 116° angle in medial view. The shaft distally expands to wrap around the medioplantar surface of metatarsal II. The trochlea faces plantarly, so that the articular surface of the trochlea is oriented perpendicular to the lateral surface of the metatarsal I that articulates with metatarsal II. In plantar view, the long axis of the trochlea is angled proximolateral-mediodistally. The distal margin of metatarsal I is located above the proximal margin of the metatarsal II trochlea. The metatarsal II trochlea is weakly angled laterodistally, and the condyles are weakly developed and widely separated. The metatarsal III trochlea is slightly wider, and the edges of the trochlear condyles are visible in dorsal view (not visible in metatarsal II). The medial surface is concave, whereas it is flat in metatarsal II; the lateral surface of the trochlea is concave in both metatarsals II and III. The metatarsal III trochlear condyles are equal in distal and plantar projection (lacking the medial differential projection of avisaurid enantiornithines^20^), but the medial condyle is slightly narrower. The single condyle of the metatarsal IV trochlea forms a narrow, strongly convex surface dorsally and distally but is expanded plantarly into a rounded condyle. It lacks the distinct crescent morphology (visible in distal view) that is present in some avisaurids^20^.

The first phalanx of the hallucal digit is long and slender. It is wider mediolaterally than it is dorsoventrally tall, and the plantar surface is excavated throughout the length of the bone. The claw is large and recurved with a broad, blunt flexor tubercle. The plantar surface of the distal trochlea is not as strongly demarcated from the shaft of the phalanx as in *Soroavisaurus*^20^. The penultimate phalanx of digit II is the longest and most robust in the foot, nearly twice the length of the preceding phalanx, and followed by a claw much larger than that of the hallux, with a flexor tubercle that is slightly shorter but deeper than that of the hallucal claw. The first phalanx of digit II is proximally much wider than any other phalanx. The distal articular surface is well developed on the cranial and plantar surfaces. The ventral surfaces of both non-ungual phalanges are deeply excavated, as in the hallux. This excavation is weakly present in digits III and IV and becomes less pronounced distally (also true of digit II). The non-ungual phalanges of digit II are asymmetrical with their medial halves showing greater plantar projection than their lateral halves. The distal trochlea is strongly expanded onto the plantar surface in the penultimate phalanges of digits II and III, indicating strong plantar flexion (grasping ability). The penultimate phalanx of digit III is the longest of this digit; it is subequal to the first phalanx of digit I and it has three-quarters of the length of the penultimate phalanx of digit II. The proximal phalanges of digit III are subequal and approximately three-quarters of the length of the penultimate phalanx. The non-ungual phalanges of this digit become increasingly gracile distally. Only a fragment of the ungual phalanx of this digit is preserved. The proximal three phalanges of digit IV are short and subequal, with well-developed proximal and distal articular surfaces. The plantar lip of the proximal articular surface is strongly developed in the proximal two phalanges. The penultimate phalanx is longer and more gracile. The proximal half of the ungual phalanx is preserved; it is more mediolaterally compressed (i.e., triangular in cross section), as in the hallucal claw, compared to the robust claw of digit II. Ligamental pits on all non-ungual pedal phalanges are poorly demarcated in the CT-scans. The ungual phalanges all preserve the horny sheaths intact (Fig. 1D,F) so that the morphology of the encased unguals (e.g., the ligamental grooves) is not visible.

DIP-V-15105b is a partial fragment of right wing found as a syninclusion alongside DIP-V-15105a, and prepared as a separate amber piece, to obtain clear views (Fig. S1). The wing and foot almost certainly belonged to the same individual. However, much of the wing is truncated at the polished surface of the amber (due to its original preparation as jewelry), creating a preservational window that does not include any additional skeletal material.

### Integumentary structures

#### DIP-V-15105a (foot)

The foot inclusion displays a range of integumentary structures that include contour feathers, scutellae scale filaments (SSFs), and unfeathered scutes and scutellae (Figs 1A–C; 2A,B). Contour feathers are present along the metatarsal tract, and extend across the bases of digits III and IV, overarching the foot far enough to reach the ungual base in digit IV (Figs 1C, S2A). Although the contour feathers are pennaceous, the barbs are loosely connected, and the barbules appear simple and blade-shaped, without differentiated flagella or hooklets (Fig. S2B). The contour feathers are preserved with a dark brown overall colouration. However, at high magnifications it is possible to discern paler central regions within the barbs, and minor banding patterns within the barbules, where their diffuse pigmentation is concentrated slightly toward the center of each internode. The visible colours preserved within this specimen appear to reflect the distribution of melanosomes within the feathers^21^, but the effects of taphonomy remain unclear. Unlike some of the other specimens from this deposit that have been sampled for melanosomes^6^, it is not possible to damage DIP-V-15105a to obtain flakes for SEM analyses.

Both scutes and scutellae have elongate SSF feathers originating from their distal margins (Figs 2A–D; S2D), similar to those observed in HPG-15-1^8^. The observable SSFs range in length from 1.53 mm to less than 1 mm long, and the broadest observed filaments are ~0.03 mm wide near their bases. The SSFs can be tracked to their insertions within follicles, excluding alternative interpretations such as fungal hyphae. The lack of preserved pigmentation in these structures suggests that they were either white or pale in life. This coloration may lead to an underestimation of their abundance and dimensions within the amber and makes these structures very unlikely to be spotted in compression fossils^22,23^. In general, filaments are longest adjacent to the unguals of digits I and II, and they occur in a dense arrangement on the dorsal surface of digits I–III. The larger SSFs appear relatively rigid and blade-shaped. They are consistently recumbent upon the surfaces of the toes, with an oblong cross-section and limited deflections due to resin flows. The filaments taper gradually to a fine point, but observation is hindered by translucency. Some SSFs are preserved with a granular texture, but this is a taphonomic artifact related to pyrite deposition^3,5^ along the outer surface of the feathers, where they have withdrawn from the surrounding amber (Fig. 2D). Scutes are only present as a narrow row along the outer surface of the digits near their distal ends; ovoid scutellae seem to replace the scutes toward the base of each digit, covering the lateral surfaces of each digit and most of the visible regions of the metatarsus (Figs 1A–C; 2A–C; S2A,D). The withdrawal of the skin from the amber surface, coupled with the contour feathers covering the basal part of the foot, may have reduced the apparent extent of the scutes. Unfeathered, circular reticulae are present along the plantar surface of each digit, together with moderately prominent digital pads. The skin of the foot has withdrawn from the surrounding amber throughout much of the reticulate area, leaving an indistinct boundary within the surrounding amber (Fig. 2A,B).

#### DIP-V-15105b (wing fragment)

The wing fragment in DIP-V-15105b captures a limited section through the overlapping primaries of the right wing (Fig. 2F–H). The rachises of ten remiges are aligned subparallel to one another, visible in cross section (Fig. 3D,E). The profile of each rachis transitions from a D-shaped base (with angular expansions adjacent to the barb rami bases), to a more sub-cylindrical profile near the apex (expanded dorsoventrally and contracted laterally). Full feather outlines are not observable due to truncation at the base and apex of each feather at the polished edge of the amber piece. However, the preserved sections of each vane show strong asymmetry. Barbs in the anterior (leading edge) vane are approximately 0.45 times as long as the corresponding barbs in the posterior (trailing edge) vane within primary 10, and the anterior barbs diverge from the rachis at ~25° while the posterior barbs diverge at ~48°. Barb rami are deeply blade-shaped. Proximal barbules within primary 10 diverge from the barb at ~37° and are relatively straight and blade-shaped; distal barbules diverge from the barb at ~58° with a deep basal blade and a weakly developed pennulum that curves strongly adapically and bears expansions and traces of hooklets at its nodes (Fig. 2F–H). The strong asymmetry, and the angles formed by the barbs suggest that the wing in DIP-V-15105 belonged to an advanced flying bird comparable to the Enantiornithes and more advanced crown-ward birds that have been analyzed^24^.

### Taxonomic and ontogenetic assessment

DIP-V-15105 is referred to Enantiornithes based on the shape of metatarsal I and metatarsal IV, and the overall configuration of the metatarsus. Differences in pedal proportions and ungual morphology indicate that DIP-V-15105 and the recently described hatchling (HPG-15-1^8^) are not referable to the same taxon, despite SSF tarsal plumage being present in both specimens. With the limited body region preserved, little comment can be made regarding ontogenetic stage of DIP-V-15105. However, we infer that this specimen is not a young juvenile, based on the well-developed articular surfaces of the metatarsals and phalanges.

The 9–10 mm long basal fragments of primary feathers preserved in DIV-V-15105b appear to be wider than those recovered in previously described specimens, with a maximum P10 width of ~5.8 mm, compared to ~4.3 mm (P9, DIP-V-1500^5^), and ~4.0 mm (P4, DIP-V-15102^9^, although barbs are truncated). However, with the limited regions of primaries available for comparison, the slightly larger feathers provide only weak support for a more developed individual. Differences in the number of primary feathers (ten in DIP-V-15105 compared to nine in both juvenile wings DIP-V-15100 and DIP-V-15101^5^), seem to indicate that the new specimen is not conspecific with either of these samples that preserve a complete set of primaries, although this difference could be ontogenetic. The lack of plumulaceous feathers on the foot, and the dense cover of contour feathers that overarch the outer toes may suggest a more mature individual. Fully developed contour feathers are an adult (basic) plumage feature in the crural tract of extremely precocial modern megapodes like the brush-turkey^25^, and differ strongly from the neoptile plumage found in HPG-15-1, an enantiornithine hatchling from Burmese amber^8^. The sample set in Burmese amber is slowly expanding in taxonomic diversity, but distinctions between groups are complicated by the range of ontogenetic stages and body regions represented by the available specimens.

### Taphonomy and behaviour

The foot morphology found in DIP-V-15105a is consistent with an arboreal, perching lifestyle^26^. The surrounding amber is composed of numerous thin flows (Fig. S1) or *Schlauben*^27^, which are associated with secretion directly on a tree trunk. Features that are associated with resin secreted near the forest floor^28^, including wood particulates and litter-dwelling insect inclusions, are absent in DIP-V-15105. Taken together, these characteristics suggest that the enantiornithine lived higher up within the resin-producing forest, and that fragments of its corpse were encapsulated in resin above the forest floor.

The DIP-V-15105 foot and wing syninclusions were originally found in close association with one another, separated by approximately 3 mm of amber. UV observations of flow lines within the amber (Fig. S1) indicate that both inclusions were affected by the same resin flows: eddies created by resin flowing between the wing feathers also swirl around the foot (Figs 3E; S2A–D). The wing feathers are oriented and overlapped in a fashion that indicates attachment to skeletal components of a wing at the time of burial—they converge toward digits and a carpometacarpus that were probably destroyed as part of the mining or polishing process. The feathers must have been held in place by skeletal or dermal (i.e., patagia) material during resin entrainment, because flow lines (Fig. 3E) clearly indicate that resin was forced to flow between the primary feathers. Polymerization prior to any significant compaction appears to have preserved the relative position of the foot and wing, also preventing distortion or shattering of the foot bones.

Bone preservation is exceptional within the foot, with all elements preserved in articulation, and the texture of osteon structures faintly visible within the exposed bone of the broken metatarsals (Figs 1D–G; 3A,C). Where polishing has crosscut the unguals, there is a distinct banding pattern to the exposed material: the ungual sheath is surrounded by a thin layer of cloudy amber (related to moisture or decay products interacting with the surrounding resin); the ungual sheath retains its original 3D structure, and is preserved as a thick, carbonized layer^29^; and the underlying ungual displays similar porosity to the bone of the broken metatarsals, overprinted by dark staining (Fig. 3B).

Jagged greenstick fractures and movement of the fractured metatarsal ends relative to one another suggests that the break occurred before the foot entered the resin (Figs 1D–G; 3C). Overall, the frothy amber veil, thick layer of carbonized soft tissues, and decay products that emanate from the broken end of the foot suggest that the specimen was still moist when it entered the resin^27^. The skin of the toes appears to have subsequently withdrawn from the surrounding resin, due to soft tissue drying while entombed (leaving an indistinct margin along sections of the plantar surface in digits I and II (Fig. 2A,B).

The combined preservational features of the foot suggest that it was torn from the rest of the body prior to entering the resin. This may relate to predation or scavenging prior to significant drying. The foot then dried within the resin but retained much of its original structure. Although the foot has almost certainly been removed from the corpse, incomplete basal preservation of the right wing precludes inferences about this additional body component. The wing may have been part of a more complete carcass prior to mining and polishing of the amber piece, or it too may have been stripped from a larger carcass.

## Discussion

### Enantiornithine development

Aside from the number of primaries that are present, the preserved wing plumage in DIP-V-15105 does not differ significantly from the juvenile enantiornithine wings previously observed in Burmese amber. Precocial feather development within this group limits the value of comparisons between the new specimen and previous finds^5,12^.

The enlarged SSFs observed in DIP-V-15105a are a puzzling feature. Their relative prominence may be attributable to interspecific variation, or their enlargement may suggest that SSFs become more pronounced with increasing maturity, given that DIP-V-15105a does not appear to be a very young juvenile. If the latter hypothesis is correct, this differs from the modern bird feet that we have been able to examine, where SSFs are rarely observed in hatchlings and seem to be absent in adults, thus limiting comparisons to enantiornithines^8^. Modern bird embryos can be manipulated to produce a larger number of prominent filaments that are more directly comparable to those in DIP-V-15105a, by introducing retinoic acid, β-catenin^30,31^ or bromodeoxyuridine^32^, or by modifying the expression of *Spry2* or *Sox18*^33^. The most similar results to date have been obtained through retinoic acid treatments and *Sox18* manipulation, which yield filaments that are virtually indistinguishable from SSFs, except for their concentration on the scutes and scutellae of the metatarsals, as opposed to the digits themselves in DIP-V-15105a.

### Integumentary structure implications

Metatarsal feathers are rare among Enantiornithes (e.g.^34^); contour feathers are more commonly found stemming from the tibia^35,36^. More basal taxa, such as *Sapeornis*, *Archaeopteryx*, *Anchiornis*, and *Microraptor* also possess pennaceous feathers of various lengths projecting from the metatarsus^37–40^. However, in these compression fossils, preservation and interpretation of smaller-scale and non-rigid feathers is limited by taphonomy. Hindlimb feathers are rarely preserved in compression fossils and it has been proposed that their phylogenetic distribution may be greater than inferred from specimens from the Jehol biota alone^39^. DIP-V-15105 provides additional data indicating that the metatarsus of at least some enantiornithines retained pedal plumage in the form of loosely vaned contour feathers and SSFs that are inclined upon the surface of the metatarsus and digits. The contour feathers are short and loosely vaned, differing structurally from the elongate and remex-like metatarsal feathers that occur erect upon the lateral surface of the foot in some Eumaniraptora taxa basal to *Sapeornis*^40^, and in some selectively bred modern Neornithes^33,41^. However, the pterylosis of the contour feathers in amber is consistent with the feather placement observed in Neornithes, and slightly more extensive than the ankle plumage of *Sapeornis*. DIP-V-15105 displays contour feathers that only cover the outer two toes. This may be a functionally constrained feature (e.g., providing clear access to the toes for the beak during feeding), but it may also be speculated to be a progressive reduction in rigid feathers (beginning with the primitive condition of remex-like feathers on the outer edge of the foot in more basal Eumaniraptora)^39^. The combined pedal plumage of enantiornithines such as DIP-V-15105 invites functional comparisons to that of modern birds, such as owls and ptarmigans, where the feathers may aid in snowshoeing, insulation, or prey capture^34^. These comparisons are complicated by the comparatively low density of SSFs on the digits, and the localized covering of contour feathers near the bases of the outer digits in the fossil specimen (compared to the extensive covering of contour feathers on all but the plantar surface of modern relatives). These differences, combined with a small body size, suggest that the pedal plumage in DIP-V-15105 may have been more useful in a tactile or role or in the handling of small prey items, such as insects.

Both the contour feathers and SSFs preserved in DIP-V-15105 lack the size and coarse structures required to have a high preservation potential in compression fossils, where they would be recorded as carbon films or impressions. Amber may provide the best medium for studying exceedingly small feather types. The presence of feathers such as these on feet in amber (in DIP-V-15105 and in HPG-15-1^8^) suggest that primitive, filamentous feather morphotypes persisted much closer to the clade containing crown group birds than originally thought^18^.

At present, the relationship between SSFs and the filamentous feathers that have been found among the tail plumage of Enantiornithes^8^, as well as the coarser filamentous ‘protofeathers’ that have been recovered from a wide range of theropod dinosaurs^18,19,42,43^, remains unclear. If SSFs are found to be directly comparable to the larger filaments, they may hold some potential for future research on the origin of feathers. Similarities between SSFs and the bristles observed in the hatchlings or developmentally manipulated embryos of modern birds^30,33,41,44^ suggest that the potential to produce these feathers is retained within crown group birds. Ultimately, conservation of this feature may allow the study of fine structural and developmental details of primitive filamentous feathers in a modern setting.

## Material and Methods

### Material and photography

DIP-V-15105 comes from the Angbamo site amber mine, Tanai Township, Myitkyina District, Kachin Province in northern Burma (Myanmar). The original specimen is housed in the Dexu Institute of Palaeontology (=DIP), Chaozhou, China. The dimensions of the original amber piece are 14 mm × 22 mm × 10 mm, and 1.69 g in weight. Specimen DIP-V-15105 began as a single piece of amber, but the relative positions of the wing and foot fragments found within the piece blocked key anatomical views. The specimen was embedded in mineralogical grade epoxy (Epo-Tek 301) under vacuum, to fill fractures that risked damaging the inclusions, and was later cut by hand with a razor saw, and re-polished to provide clear views of both inclusions. DIP-V-15105a refers to the resulting amber piece containing the foot, while DIP-V-15105b contains the wing fragment.

Inclusions were examined using a Leica MZ 12.5 dissecting microscope. Photographs were taken using a Visionary Digital imaging station that consists of a Canon camera (5D Mark III, with MP-E 65MM F/2.8 1-5X lens) fitted to a macro rail (Cognisys) that captures images at multiple focal planes. Images were processed using Helicon Focus 5.1 and Adobe Photoshop CS5 software to increase depth of field. Images were taken under transmitted light, with dark field illumination (d.f.) to image translucent structures, and with long wavelength UV light to map resin flows.

### Burmese amber

‘Burmese amber’ refers to a regional deposit in the Hukawng Valley of the Kachin State, in northern Myanmar. The deposit has been assigned a late Albian–Cenomanian age (~105 to 95 Ma) based upon ammonite and palynomorph fossils in the surrounding strata^45^. This estimate has been refined to approximately 98.8 ± 0.6 Ma, based upon U-Pb dating of zircons in the matrix surrounding the amber^3^. The deposit is thought to have been produced by trees belonging to either the Cupressaceae or Araucariaceae, that lived in a moist tropical setting^1,2^. The faunal contents of the deposit have been the source of intense research activity recently, which has been reviewed^41,46^; meanwhile, general features of the deposit are covered in the syntheses of Grimaldi *et al*.^1^ and Ross *et al*.^2^.

### Micro-CT scanning and 3D reconstruction

Specimen DIP-V-15105a was imaged using propagation phase-contrast synchrotron radiation microtomography (PPC-SR-µCT) on the beamline 13 W at the Shanghai Synchrotron Radiation Facility (SSRF). The isotropic voxel size is 3.25 mm. The beam was monochromatized at an energy of 25 keV using the double crystal monochromator. Amira 5.4 (Visage Imaging, San Diego, USA) was used to reconstruct the specimen osteology, based on the image stacks obtained. VG Studiomax 2.1 (Volume Graphics, Heidelberg, Germany) was used to complete volume renderings and animations, while final figures were prepared with Photoshop and Illustrator CS5 (Adobe, San Jose, USA).

### Terminology

Integumentary structure terminology generally follows that of Lucas and Stettenheim^47^ (including tarsal scale types), Dove^21^, O’Connor^35^, and Xing *et al*.^8^.

## Supplementary information

Supplementary Information

## Data Availability

All data are available in the main text or supplementary materials.
