# Supplementary material for: A fully feathered enantiornithine foot and wing fragment preserved in mid-Cretaceous Burmese amber
Source: Sci Rep. 2019 Jan 30;9:927. doi: 10.1038/s41598-018-37427-4 (PMC6353931; doi:10.1038/s41598-018-37427-4)
Supplement: Supplementary file 1 — Supplementary Information [file 41598_2018_37427_MOESM1_ESM.pdf]

Supplementary Information

A fully feathered enantiornithine foot and wing fragment preserved in mid-Cretaceous Burmese amber

Lida Xing[1 2,†], Ryan C. McKellar[3,4,5,†*], Jingmai K. O'Connor[6,†*], Ming Bai[7], Kuowei Tseng[8], Luis M. Chiappe[9]

1. State Key Laboratory of Biogeology and Environmental Geology, China University of Geosciences, Beijing 100083, China
2. School of the Earth Sciences and Resources, China University of Geosciences, Beijing 100083, China
3. Royal Saskatchewan Museum, Regina, Saskatchewan S4P 4W7, Canada
4. Biology Department, University of Regina, Regina, Saskatchewan S4S 0A2, Canada
5. Department of Ecology & Evolutionary Biology, 1501 Crestline Drive – Suite 140, University of Kansas, Lawrence, Kansas 66045, USA
6. Key Laboratory of Vertebrate Evolution and Human Origins of the Chinese Academy of Sciences, Institute of Vertebrate Paleontology and Paleoanthropology, Beijing 100044, China
7. Key Laboratory of Zoological Systematics and Evolution, Institute of Zoology, Chinese Academy of Sciences, Beijing, 100101, China
8. Department of Exercise and Health Science, University of Taipei, Taipei 11153, China
9. Dinosaur Institute, Natural History Museum of Los Angeles County, Los Angeles, CA 90007, USA

*** Contact information:** jingmai@ivpp.ac.cn (J.K.O.); ryan.mckellar@gov.sk.ca (R.C.M.).

[†] These authors contributed equally to this work.

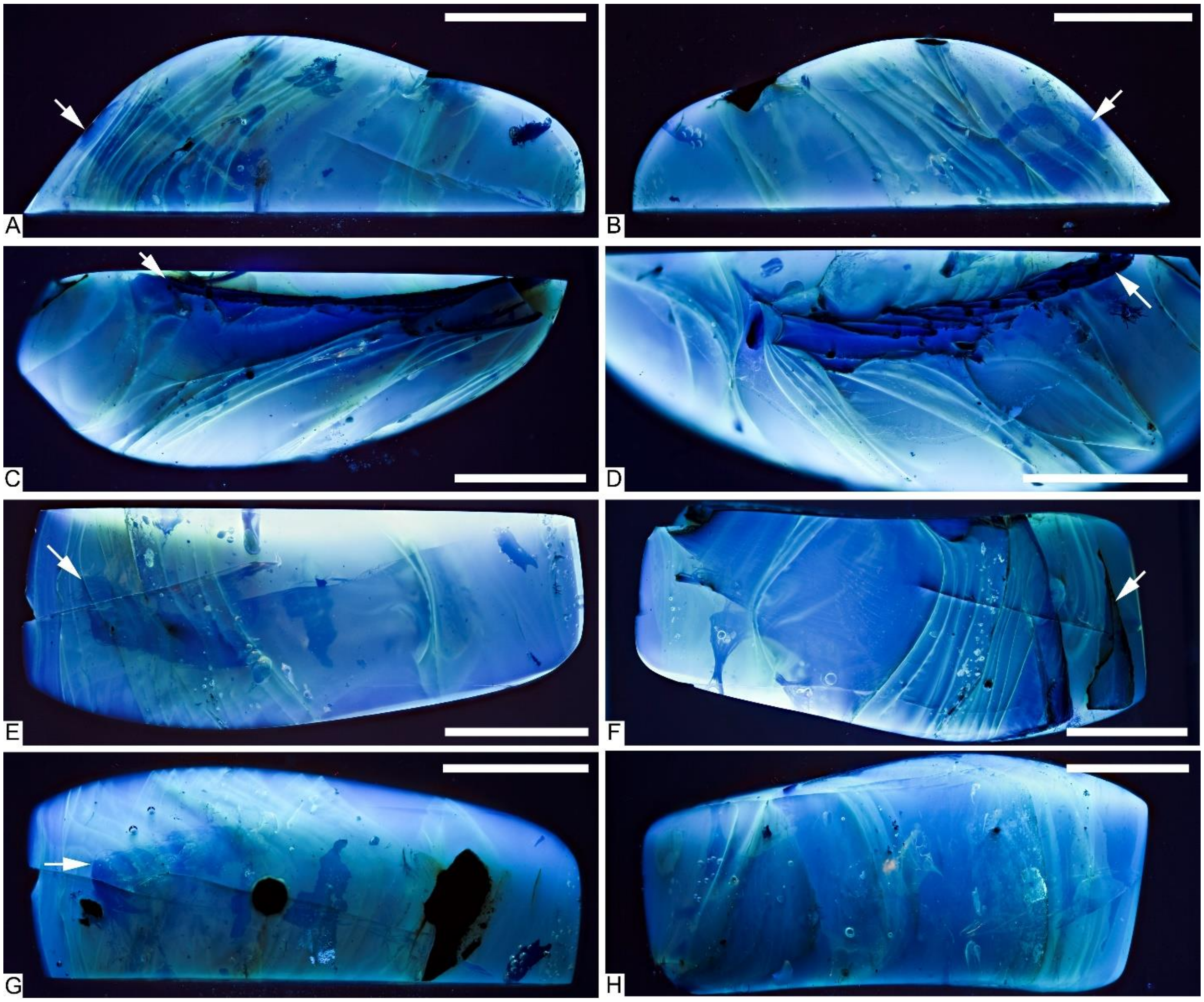

Figure S1 ｜ UV images of flow lines and syninclusion orientations in DIP-V-15102a foot, and DIP-V-15102b wing.

(A) DIP-V-15102a, foot in predominantly lateral view, flat lower surface is cut between (A) and (C), where approximately 1 mm of amber was removed during cutting. **(B)** DIP-V-15102a, foot in predominantly medial view, flat lower surface is cut between (B) and (D). **(C)** DIP-V-15102b, wing feathers in apical view, flat upper surface connects with (A). **(D)** DIP-V-15102b, wing feathers in basal view, flat upper surface connects with (B). **(E)** DIP-V-15102a, foot in predominantly dorsal view, along cut between two inclusions. **(F)** DIP-V-15102b, wing feathers in ventral view, along cut between two inclusions. **(G)** DIP-V-15102a, foot in predominantly plantar view. **(H)** DIP-V-15102b, wing feathers in dorsal view. Arrows point to apex of digits in foot (A, B, E, G), and leading edge of wing (C, D, F); flat surface between paired views (A and C, B and D) is cut line in amber that separated foot from wing inclusions. Scale bars = 5 mm.

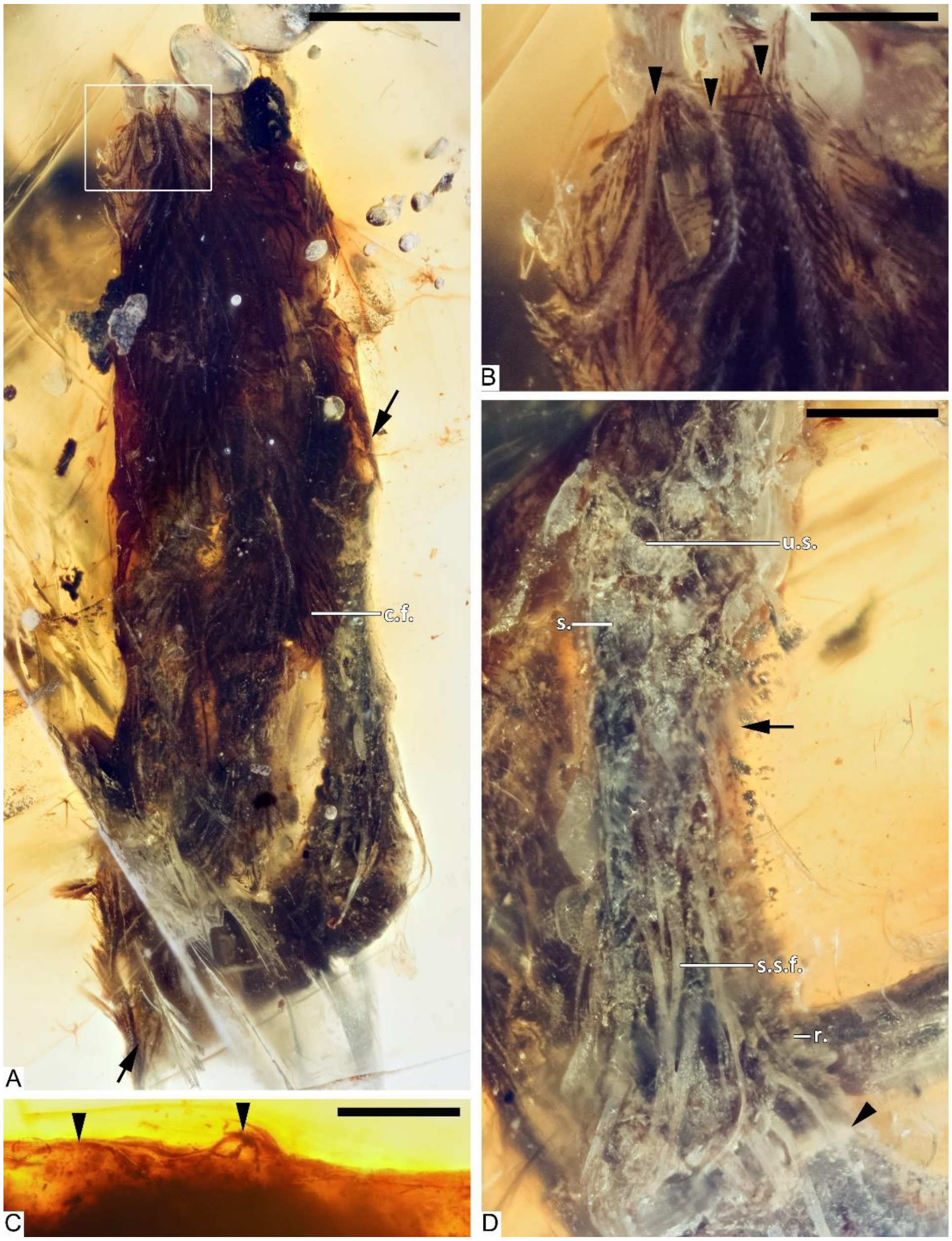

Figure S2 ∣ Additional details of integumentary structures in DIP-V-15102a.

(A) Higher magnification view of dorsal surface of foot, with severed ends of metatarsals at top, and unguals at bottom of image; arrows mark apicalmost extent of contour feathers covering outer toes; box indicates magnified area in B; and c.f. indicates apex of single contour feather. **(B)** Details of barbs and barbules in contour feathers near base of foot, with arrowheads marking unpigmented barb rami and basal sections of barbules in barbs that otherwise have dark brown apparent coloration. **(C)** Detail of unfeathered skin apically in digit III, with scutellae or reticulae indicated (arrowheads). **(D)** Details of SSFs, scutes, and scutellae on dorsal surface of digit II, with base of toe toward top of image; transition from SSF-dominated integument to oblong scutellae occurs near arrow, and SSFs become more pronounced and numerous toward base of ungual (arrowhead). Abbreviations provided in Fig. 1. Scale bars = 1.0 mm (A); 0.25 mm (B, C); 0.5 mm (D).
